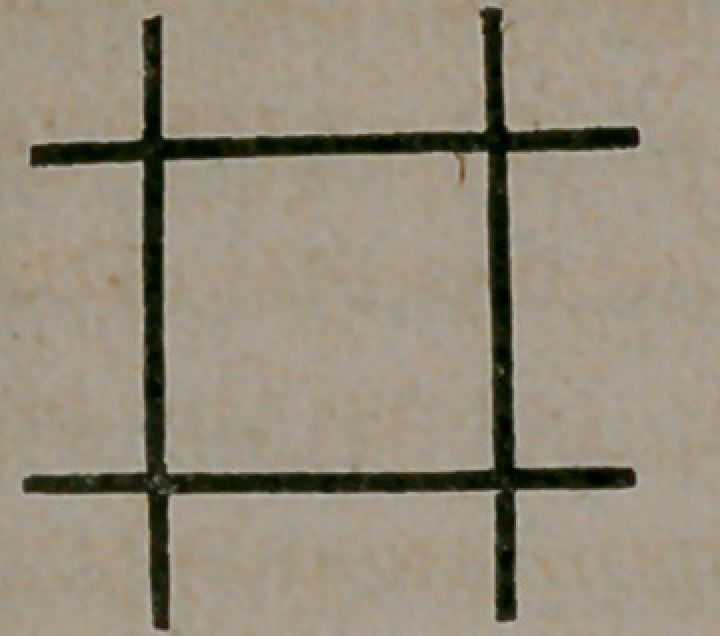# The Improved Galvano-Puncture Needles

**Published:** 1873-03

**Authors:** Alex. Murray

**Affiliations:** New York


### THE IMPROVED GALVANO-PUNCTURE NEEDLES.

### *THEIR CONSTRUCTION, USE, AND MODE OF APPLICATION, Etc.*

BY ALEX. MURRAY, M.D., NEW YORK.

The galvano-puncture needle I have nominally divided into three parts—namely, the point, the free metallic surface or conductor, and the insulated portion.

The point of the needle should be made sufficiently sharp to cut its way gradually through whatever tissues desired without lacerating them. The lance or spear-shaped point should increase in width and thickness gradually, from the extreme point to the shoulder. The latter should always be wider transversely than the stem or shaft. The bayonet point should have its three cutting edges sharp and tapering, and, where it joins the stem proper, the same diameter. The round point is not suitable for large needles; for small ones it answers very well. This form of point always forces an entrance by displacement, or by circular compression, rather than by an ample clean incision. The free metallic portion of the needle should be either cyllindrical or flat in shape, and sufficiently large to convey electricity wherever desired. The insulated portion of the instrument should be well covered with a good insulating material, so that the electric current cannot escape except where directed.

In manufacturing needles for electrolytic purposes, the part specially intended to be insulated should be smaller cylindrically than the adjoining portion of the stem, so that when the insulation is properly applied, the instrument will present a smooth level surface with the corresponding part of the needle. The stem proper of the needle, when complete and ready for use, should have a uniform diameter throughout its whole length. The best material to use for insulating needles is hard rubber applied *secundum artem.* No other simple substance, or any combination of materials yet discovered, can supply the place of rubber for durability or perfection of insulation.

The galvano-puncture needles present a different arrangement of the insulation from any that are now in use. The point of the needle is free from insulation from one-quarter to one inch. The succeeding one-half or three-quarters of an inch is insulated. The next portion of the needle is the most important, and the part which I have selected to be used as a subcutaneous conductor, by leaving the metallic surface exposed from one-quarter to one or two inches, or even more, according to the length of the instrument, and the remaining part insulated to the eye.

The insulated portions of the needle are the parts which are intended to remain in contact with the skin during the operation, so as to prevent ulceration or sloughing.

The peculiar method of arranging the insulation of the needles I regard as a valuable improvement, and one suited for almost all cases where their use may be required. Every day's experience convinces me more and more of the utility of the needles, and that they are in every way better adapted for the resolution of *morbid growths, hydrocele, bursæ, nævi, hemorrhoids, varicose veins, etc.*

They can be introduced into a tumor or vein as easily as the ordinary needle in use, and have this *special advantage of retaining whatever position they may be placed in, without slipping out or loosen-*

*ing their hold under the action of the galvanic current with a negative attachment, while they present more metallic surface to be acted upon by the current, and cause less pain to the patient.* They can be used in the same manner as the needles now in use, by simply coating the middle metallic portion with white wax, collodion, or adhesive plaster.

In using needles to discuss tumors or nævi, etc., by electrolysis, there are several practical points to be observed in order to obtain satisfactory results. The needles should be made of gold, platina, well-tempered steel annealed, plain, gilt, or platinized. An alloy of platinum and iridium, or steel with a small portion of platinum, about three parts to the one hundred. This alloy of steel is frequently used in fine cutlery, and has this advantage, that it removes to a certain extent from steel its brittleness and tendency to oxidation.

The needles should be smooth and well-polished, thoroughly insulated, and with a round, spear, or bayonet point. It is essential that a limited portion of the metal should be free from any insulation, so as to allow sufficient surface to deliver the action of the current where directed. The needles should be introduced from the circumference laterally, or in any direction desired, into the middle or base of the morbid growth, from one side to the opposite.

In this way they will transfix it so completely as to leave a portion of the insulation in direct contact with the sound skin, at the entrance and emergence of the needle. It is always best to insert the needles singly and in parallelism; a slight obliquity, however, will not make much difference.

The distance of one needle from the other may vary from one-half to one inch more or less, according to the size of the morbid growth, and their action then would be sufficiently electrolytic without being too localized. Should one pole, the negative, only be used, a number of needles from one-quarter to one-third of an inch apart is probably the very best arrangement for immediate localized action.

The needles are straight and curved, and from two to six inches in length.

DIRECTIONS FOR USING THE NEEDLES.

*The first thing necessary in using the needles.*—First select the needle or needles suitable to the size of the morbid growth on which we are about to operate, and then *place the non-insulated middle portion of the needle* or needles on the diseased part where the insertion is to be made. This portion should extend over three-quarters of the diameter of the tumor, etc., or should at least allow sufficient of the insulated parts of the instrument when inserted to be in contact with the skin.

*Position of the operator and patient during the inserting or manipulation of the needles.*—The physician, before he begins the operation of inserting the needles, should choose that position both for himself and the patient in which he can work to the best advantage. He should either stand or sit opposite to the part of the disease on which he is going to operate; or on one side, should he deem such a position the most suitable. Should the case in hand require that his position should be in front, or the left side, my rule is to insert the most distant needle the first, and proceed from above downwards, or from below upwards, *seriatim,* until all the needles are in their proper position.

*Mode of insertion and caution to be taken.*—The operator should commence the operation by compressing the tumor between the thumb and fingers of the left hand, should the excrescence be large; but if it should be a small one, or with a sessile base, he should either stretch or pinch up the skin with the forefinger and thumb, so as to render it tense and steady, and then with the right hand insert the needle into the skin, keeping the point close to the thumb. After this is done, he should pass it fearlessly and deliberately through the middle or base out to the opposite side, so as completely to transfix it, letting the point emerge near the forefinger. After each needle is placed in its proper position, he ought to cover the exposed point immediately with a small piece of cork-wood to prevent injury to the patient or medical attendant, in case either of them should accidentally touch it.

*The best mode of operation in cases of large morbid growth.*—As a general rule, the best treatment for large morbid growths is to use both poles of the battery in the body of the diseased part, and allow the distance between the needles to vary from a half to one inch. By this means we can get the full influence of the current directed where it is desired, and so effect a speedy resolution of the disease by electrolysis. Should the negative pole only be used in the body of the tumor, the positive should be applied with a suitable well moistened sponge or chamois-covered electrode on the surface directly over the needle or needles, or "*vice versa.*"

*General application of the galvanic current; the best way of proceeding in electrolysis.*—For the electrolytic treatment of tumors, etc., the application of the galvanic current, either with or without general or local etherization, should always at first be mild and gradually increased to the required strength, and should never be employed until all the appliances of the needles are properly arranged, and secured directly to the battery, or by some portable arrangement for holding the needles. My "*serres fines*" will be found a convenient instrument for holding one or more needles that may be required, connected by an interrupting handle to conduct or break the current when desired. If the battery has been recently filled, from sixteen to twenty cells will be amply sufficient to employ; but if in use some time, twenty-four to thirty cells.

The conductors should be made of strands of very fine copper wire, about fifteen or sixteen pieces of No. 35, twisted into cords, and covered either with silk or cotton of two colors—red for the positive, and green for the negative pole.

*Length of séance.*—The duration of the séance for adults may range from ten to forty minutes, according to the nature and size of the diseased growth. For children, from three to ten minutes will suffice, except the operation is performed on the head or neck, when two to five minutes will be amply sufficient.

The best method of operating on *nœvi* by electrolysis is to insert the needles in the form of a triangle, as represented in the annexed wood-cut.

The engraving shows at a glance the way in which the needles should be arranged. This method possesses a decided superiority over the usual mode adopted in the arrangement of the needles around and through the base of the circumference of the vascular mass.

The same principle is here shown of insulating or strangulating the excrescence by cutting off nutrition with the needles, as would be done by the ligature subcutaneously. If the nævus is a large one, four needles may be inserted in a quadrangular form—thus:

or they may form as many sides as the number of needles employed. When operating with needles on nævi, they should be inserted *through the base of the tumor only,* allowing the entrance and exit of the needles to appear in the sound skin. A sufficient number should be employed at the same time. And never, if I may so speak, should the growth be tickled with small, inefficient needles. The object we have in view, when operating with needles, is to destroy, or at least arrest, the circulation of the blood in the vessels which supply nutrition to the excrescence. The latter undergoes, after the electrolytic action of the needles, the process of consolidation from disintegration or coagulation of the blood; wasting, and drying up of the different tissues composing the vascular growth occur, leaving the *débris,* generally in the form of a dry crust, to fall off in one or more weeks.

The electrolytic treatment of nævi possesses its chief advantage in being safe, practical, and easily performed. Better results will generally be found to follow this mode of operating than the methods usually employed. Besides, there is no danger from hemorrhage, inflammation, suppuration, or pyæmia, nor sloughing when due care is observed.

As a rule, I would forbid the use of steel or gilded steel needles attached to the positive pole of the battery, on nævi situated on any part of the face. The nascent protoxide of iron eliminated by the electrolytic action of the positive pole on steel needles will occasionally leave a permanent blue stain in the dermoid tissues after the disappearance of the nævi. I noticed that this discoloration occurred in two cases after my first operations with steel needles.

The oxides of the iron *per se* may act mechanically in arresting the flow of blood (?), but they do not possess any hæmostatic properties whatever, unless combined with a mineral acid to form a *proto* or *per* salt of the mineral.

I also make it a rule not to meddle with nævi situated on the anterior fontanel in infants under two years of age, except occasionally to make local applications of the tincture of iodine, in order to check the further growth of the excrescence.

We find that as soon as the osseous structures begin to close the opening, and frequently long before the closure of the fontanel occurs, the nævus disappears spontaneously. The reason is obvious: as the frontal and parietal bones progress in their growth towards closing the diamond-shaped opening of the head, the blood-vessels which supply the excrescence are gradually compressed by the slow progressive growth of the bones, and ultimately obliterated. The vitality of the growth of the vascular mass is thus impoverished from want of blood, and the mass itself gradually wastes away, until it finally disappears without leaving any trace to indicate where it had existed.

I have noticed the spontaneous disappearance of nævi occur in four cases—in one of them it took place in less than three weeks.

### HYDROCELE.

Before we operate for hydrocele with needles, one or two things must be observed, if the accumulation of liquid is large. I would recommend that a portion, at least one-half, of the contents of the sac be withdrawn before the needles are employed. We shall find that, by adopting this mode of proceeding, that we lessen the quantity of the fluid to be acted upon by electrolysis, and allow room for the collection and the distention of the nascent gas as it arises from the catalytic action of the needles in the liquid, and that we hasten the process of absorption. This latter process should always be allowed to take place spontaneously after electrolyzation. It may be aided, if deemed necessary, by the daily applications of the continuous current to the scrotum externally, in a labile manner, by means of well-moistened sponge-covered electrodes. The latter should never be allowed to remain more than a minute on any one part. We may use three or four needles in the interior of the sac, and attach every second needle to the positive pole and the remaining needles to the negative. This arrangement works better than to have two negatives and two positives together, a proceeding which renders the action of the latter too localized.

Should we employ three needles only, with both poles in the sac, the outside needles should be connected with the negative pole, while a large needle of platinum in the middle with the positive.

Having operated with my needles in eleven cases of hydrocele by electrolysis, I can recommend with confidence the methods above suggested.

Physicians occasionally meet with disappointment when using needles in the treatment of hydrocele by means of the galvanic current. There are times and conditions of individual cases when all modes of treatment will occasionally fail. The most apparent cause of failure in electrolysis arises from the fact that we employ needles which are too small and liable to fall out of the sac during their use when connected with the negative pole. The free metallic surface of the needles that should be employed in the interior of the sac, should at least be from one and a half to two inches in length. There should never be less than two or three needles employed at any one operation. I allude particularly to my own needles; as they present a larger surface, fewer needles will be required than are now usually employed. Although my needles make two punctures—entrance and exit—yet, when properly inserted, this is a practical advantage rather than a disadvantage in most if not in all cases requiring their use for electrolysis. A needle too many, or too large, at any time, is preferable, to small, inefficient needles, or an inefficient number, in electrolysis.

Their advantage in hydrocele is apparent when there is a large quantity of liquid in the sac to undergo an electrolytic chemical change. By employing a sufficient number of needles in the interior of the sac, we change the causation of the morbid action, and restore the balance between secretion and absorption.

The electrolytic treatment of hydrocele with the needles is unattended with danger or inconvenience, and recommends itself from its great simplicity and apparent freedom from pain, and the success attending it is likely to supersede the trocar and canula with or without injections, setons, incisions in the sac, acupuncture, etc., as hitherto practiced, and not always successful.

The needles should be inserted through the upper part of the scrotum, and carried down close along its anterior and inner surface to the opposite point or bottom of the sac, and again thrust through the scrotum wherever desired. By passing the needles downwards (vertically), rather than from below upwards, laterally or obliquely, we can avoid doing any injury to the testicle. This mode of proceeding will be found not only the most convenient, but the safest and best to adopt in almost all cases of hydrocele.

*The mode of operating in hydrocele.*—Local anæsthesia is seldom required before commencing the operation with needles for hydrocele. The patient should stand against a door or wall, with his legs far enough apart, so as not to interfere with the hands of the operator. The physician should sit opposite, a little to his right, and grasp the scrotum behind with his left hand, so as to render it tense, steady and prominent anteriorly. He should also ascertain that the testicle is not in the way, so that it can be injured by the needles. The latter should be of a size suitable to the hydrocele on which he is about to operate. The needle should be held perpendicular to the scrotum, between the thumb and forefinger of the right hand. The operator should then engage the attention of the patient, and ask him when he touches the scrotum (the part selected for the insertion of the needle) with the point of the needle if it gives him any pain, and before a reply can be made let him push the instrument through the scrotum downwards into the sac, and out through the lower part of the scrotum, so as to completely transfix it, with a quick, semi-rotary motion. After the first needle is in position, he will find that the patient will not object to the introduction of the remaining needles, as the pain is comparatively trivial.

When all the needles are in an arranged position, and attached to the *serres fines*, with an interrupting handle connected to the negative pole of the battery, the galvanic current may then be allowed to flow from fifteen to twenty minutes. At first it should be mild, but afterwards gradually increased to such strength as the patient can comfortably bear. The positive pole should be applied by means of a sponge-covered disk to the scrotum directly over the needles.

The scrotum should be shaken or kneaded frequently during the séance, so as to insure the full influence and general distribution of the galvanic current through every portion of the fluid contained in the scrotal sac, and thus avoid immediate localized electrolyzation. A slight œdema and inflammation of the scrotum occasionally fellow the use of the needles. The application of a cold lotion containing hydrochlorate or acetate of ammonia to the part affected for a day or two is all the local treatment required. It is necessary, however, after the needles are withdrawn, that the patient should wear for some time a suspensory bandage to sustain the weight of the scrotum, and to afford him relief in case of any hardness or enlargement remaining after the absorption of the fluid.

Our success in operating by electrolysis depends chiefly on having a good galvanic battery, and good insulated needles. The elements of the battery should be of a medium size, neither too large nor too small, so as to allow at least nine or ten inches of surface of each element to be exposed to the action of the generating battery fluid, especially when an *acid* solution is employed. A larger amount of surface is required when we use a mineral salt dissolved in water as an exciting liquid.

What is most desirable in the battery we employ, especially for electrolysis, is an instrument possessing a large quantity of electricity, with considerable intensity to overcome resistance, and to render the electrolytic process possible. Daniels' battery, with its various modifications, is the most reliable and constant yet devised, but its great weight and bulk or want of portability confines it to hospital or office use. Bunsen's battery, with its modifications, is more portable, as is Stöhrer's, and which is still further improved by the Galvano-Faradic Company, J. Kidder, and Curt Myer. The portable batteries manufactured by the above firms are reliable, and possess all the qualities necessary for electrolysis.

In order to have a regular and reliable current, the battery should be charged with fresh, well-mixed battery fluid the day we perform any important operation. There should be the same quantity, accurately measured, of liquid in each cell, and the elements should be immersed in the cells to the same depth. I allude to the portable batteries. The interior of the binding screws, the ends of the conducting wires, and the various metallic connections, should be perfectly free from metallic oxides or dirt, and all the necessary connections should be properly secured to the battery. The latter, also, should be set in operation a few minutes before we require its use, as by this procedure we can avoid the fluctuating current generated when the elements are first immersed in the battery fluid.

A strict attention to these minor details will prevent disappointment, and insure the proper action of the battery, and success will attend our operations. Hammond's permanent hospital battery is the most reliable and constant battery in the United States. Brenner's galvano-faradic machine, as modified and improved by the Galvano-Faradic Company, is an admirable instrument. The physician who is the happy possessor of a Brenner has a treasure, and requires no other apparatus for electro-therapeutics during a lifetime. The size and weight of the cells, and the permanent fixtures, confine their use to hospital or physician's office.

Curt Myer's new portable galvanic battery, a modification of Lechanché's, is a very neat, ingenious, and well-made instrument. The small size of the elements that are used in the construction of this apparatus are not sufficient to give a large quantity of electricity with intensity suitable for electrolysis. Not having tested the merits of this instrument, I cannot speak decidedly as to its practical utility in electro-therapeutics. The galvano-cautery battery is a special instrument, and employed principally for the purposes its name implies.

Dresher's galvanic battery, with the U-shaped cells, has not in my hands answered my expectations, although I regard it as a neat and portable apparatus. The elements are too small for the battery fluid employed, and the value of the battery is sacrified for portability. The instrument will be found suitable for operations on such delicate organs as the eye, ear or urethra; but for the electrolytic treatment of diseases with the use of the needles, it will be found to lack sufficient *quantity of electricity.* In order to obtain the same result conditionally with this instrument as with any of the thirty or thirty-two cell batteries manufactured by the names already mentioned, between seventy and eighty cells would have to be employed to be equivalent in quantity or to obtain any satisfactory electrolytic results, and then, practically speaking, the *intensity* would be considerably in excess for electrolysis. In a word, this instrument gives a *minimum* portion only, instead of a moderate or proportionate share of quantity of electricity for the number and size of the elements employed.

The following case of nævus shows the result of the action of two different batteries:

Dr. G. Thompson wished me to operate on a child eleven months old, who had a subcutaneous erectile tumor, about the size of a walnut, situated on the left side of the vulva. The nævus, a few days previous to my visit to the patient, had been ruptured. This having occurred some time during the night, a considerable quantity of blood was lost before the accident was discovered. I used general anæthesia, and inserted three of my needles, in the form represented in the wood-cut. I attached each needle to the negative pole of the battery, closing the circuit directly over the tumor with a small platina disk, and then employed a current of twelve cells of Dresher's battery, charged with fresh battery fluid that day, and increased gradually to twenty, the full amount of the battery. The séance lasted twenty minutes without any marked result. Seven days after the operation, the blood-vessel which had been previously ruptured again gave way, and hemorrhage occurred to a considerable extent before it could be arrested. I felt somewhat disappointed at the result of this operation with the needles; but I found that the fault was in using a battery current *deficient in quantity*, and and not in the needles.

As the child was weak from loss of blood, I went prepared to strangulate the nævus with the ligature subcutaneously. Dr. T. was anxious that I should test the needles by the use of another battery. After local anæsthesia, I inserted three needles in the same manner as in the former operation, and employed a current of ten cells of the zinc-carbon manufactured by the Galvano-Faradic Company. The current was employed for ten minutes. In a few moments a marked change was visible. The purplish-red color of the nævus soon changed to a pale leaden hue, as if the part had been suddenly frozen, and also presented a hard, shriveled appearance. In less than three weeks a brown, dry crust fell off, leaving a small cicatrix to show where it had existed.

I have operated in a considerable number (seventeen) of cases of nævi with my needles, and have obtained satisfactory results. In five of the cases, especially in large mixed nævi, I have had to make two—and in one of the cases three—applications of the needles before I could arrest the vitality of the excrescence. I must say that this occurred before my present improved method of arranging the needles. With the present arrangement, a second application will not often be required.

I look upon the galvano-puncture needle, when employed in a tumor, and attached to the negative pole of a galvanic battery in operation, as similar in its effects to a mitrailleuse or Galting gun on a small scale discharging its bullets into the camp of the enemy, but comparatively more powerful and destructive, as it explodes its *miniature bomb-shells of nascent hydrogen* into the tumor, causing disintegration of the different tissues directly engaged, both by their mechanical or chemical action, separately or combined.

The galvano-puncture needles can be had from the Galvano-Faradic Company, 167 East Thirty-fourth street, New York.

239 East Tenth Street.

## Figures and Tables

**Figure f1:**
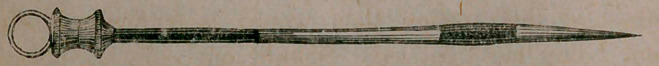


**Figure f2:**
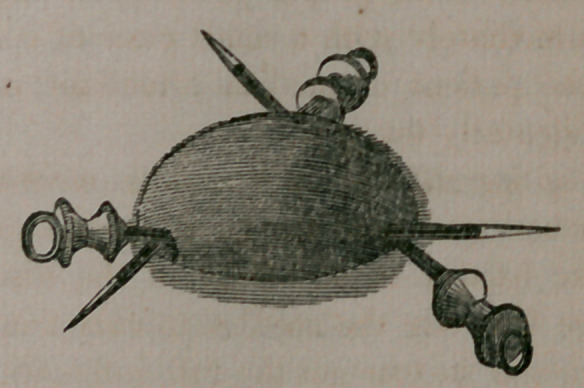


**Figure f3:**